# Surgical Advances in Parkinson’s Disease

**DOI:** 10.2174/1570159X21666221121094343

**Published:** 2023-04-27

**Authors:** Victor S. Hvingelby, Nicola Pavese

**Affiliations:** 1 Department of Clinical Medicine, Nuclear Medicine and PET Center, Aarhus University, Aarhus, Denmark;; 2 Clinical Ageing Research Unit, Newcastle Upon Tyne, Newcastle University, United Kingdom

**Keywords:** Deep brain stimulation, MRgFUS, movement disorder, neuromodulation, neurosurgery, Parkinsons

## Abstract

While symptomatic pharmacological therapy remains the main therapeutic strategy for Parkinson’s disease (PD), over the last two decades, surgical approaches have become more commonly used to control levodopa-induced motor complications and dopamine-resistant and non-motor symptoms of PD. In this paper, we discuss old and new surgical treatments for PD and the many technological innovations in this field. We have initially reviewed the relevant surgical anatomy as well as the pathological signaling considered to be the underlying cause of specific symptoms of PD. Subsequently, early attempts at surgical symptom control will be briefly reviewed. As the most well-known surgical intervention for PD is deep brain stimulation, this subject is discussed at length. As deciding on whether a patient stands to benefit from DBS can be quite difficult, the different proposed paradigms for precisely this are covered. Following this, the evidence regarding different targets, especially the subthalamic nucleus and internal globus pallidus, is reviewed as well as the evidence for newer proposed targets for specific symptoms. Due to the rapidly expanding nature of knowledge and technological capabilities, some of these new and potential future capabilities are given consideration in terms of their current and future use. Following this, we have reviewed newer treatment modalities, especially magnetic resonance-guided focused ultrasound and other potential surgical therapies, such as spinal cord stimulation for gait symptoms and others. As mentioned, the field of surgical alleviation of symptoms of PD is undergoing a rapid expansion, and this review provides a general overview of the current status and future directions in the field.

## 1 INTRODUCTION

Before the advent and introduction of levodopa therapy to treat Parkinson’s Disease (PD) in 1961 [1], surgery was considered an indispensable treatment. However, as surgery showed inconsistent and inferior results, not to mention a substantially increased risk of adverse events compared to levodopa, surgery was discouraged for PD treatment. Over recent decades, interest in surgical treatments for PD has resurfaced, most notably with the introduction of Deep Brain Stimulation (DBS) in the 1990s. Since then, neuromodulatory surgery has steadily been gaining traction with a concomitant increase in current treatment options and ideas for future surgical strategies. In this narrative review, we will focus on some of the advances that have been made in the field of the surgical management of PD since the introduction of DBS. The evolving knowledge of existing therapies, their efficaciousness in treating different symptoms of PD, future directions as well as novel treatment strategies will also be discussed. A brief introductive description of the anatomy and physiopathology of the basal ganglia will also be provided for a better understanding of the brain targets for surgery and the onset of potential side effects, most of which are due to anatomical proximity with non-target structures or non-motor intra-target structures.

## 2 SURGICAL ANATOMY OF BASAL GANGLIA – A BRIEF REVIEW

The basal ganglia, or basal nuclei, are responsible for several functions, including integration of motor control, sensorimotor integration, motor learning, and executive functions. The basal ganglia consist of the dorsal striatum, subdivided into the putamen and caudate, the globus pallidus (GP), subdivided into pars externa and pars interna (GPe and GPi, respectively), the subthalamic nucleus (STN), and the substantia nigra (SN), subdivided into pars compacta (SNc) and pars reticulata (SNr).

The primary inputs to the basal ganglia are received by the striatum from different parts of the cortex. The dorsal striatum is anatomically divided into medial and lateral parts. This division corresponds to the caudate and the putamen, and though divided by the internal capsule, they are functionally a single nucleus. The medial part, the caudate, is a paired structure near the center of the brain, sitting just rostral to the thalamus and with the head and corpus comprising part of the floor of the lateral ventricle. It winds its way caudally in a sigmoid fashion, and the final part is positioned in an anteriorly pointing fashion. The posterolateral part of the dorsal striatum (or the putamen) is a rounded structure, forming the most lateral pole of the basal nuclei. As mentioned, it is continuous with the caudate, extending from the anterior end. Rostral to the putamen is the radiation of the internal capsule. The GP is located along the medial side of the putamen. Together, these two structures are sometimes referred to as the lentiform nucleus.

Inferiorly to the thalamus and macroscopically continuous with it, there is a small collection of grey matter called the zona incerta (ZI). The ovoid STN is just anteroinferior and slightly ventral to the ZI [2]. The continuum between the STN and ZI makes it difficult to distinguish these two structures intraoperatively [3, 4], an anatomical proximity that, however, can be potentially useful for the treatment of parkinsonism [5]. The red nucleus is located just medially, adjacent to the midline, and slightly posteroinferior to the STN. The SN stretches inferiorly to these two nuclei. The STN is separated laterally from the GPi by the collection of pallidofugal fibers known as the ansa lenticularis [6].

## 3 NORMAL CIRCUITRY OF THE BASAL GANGLIA

**Fig. (1):**
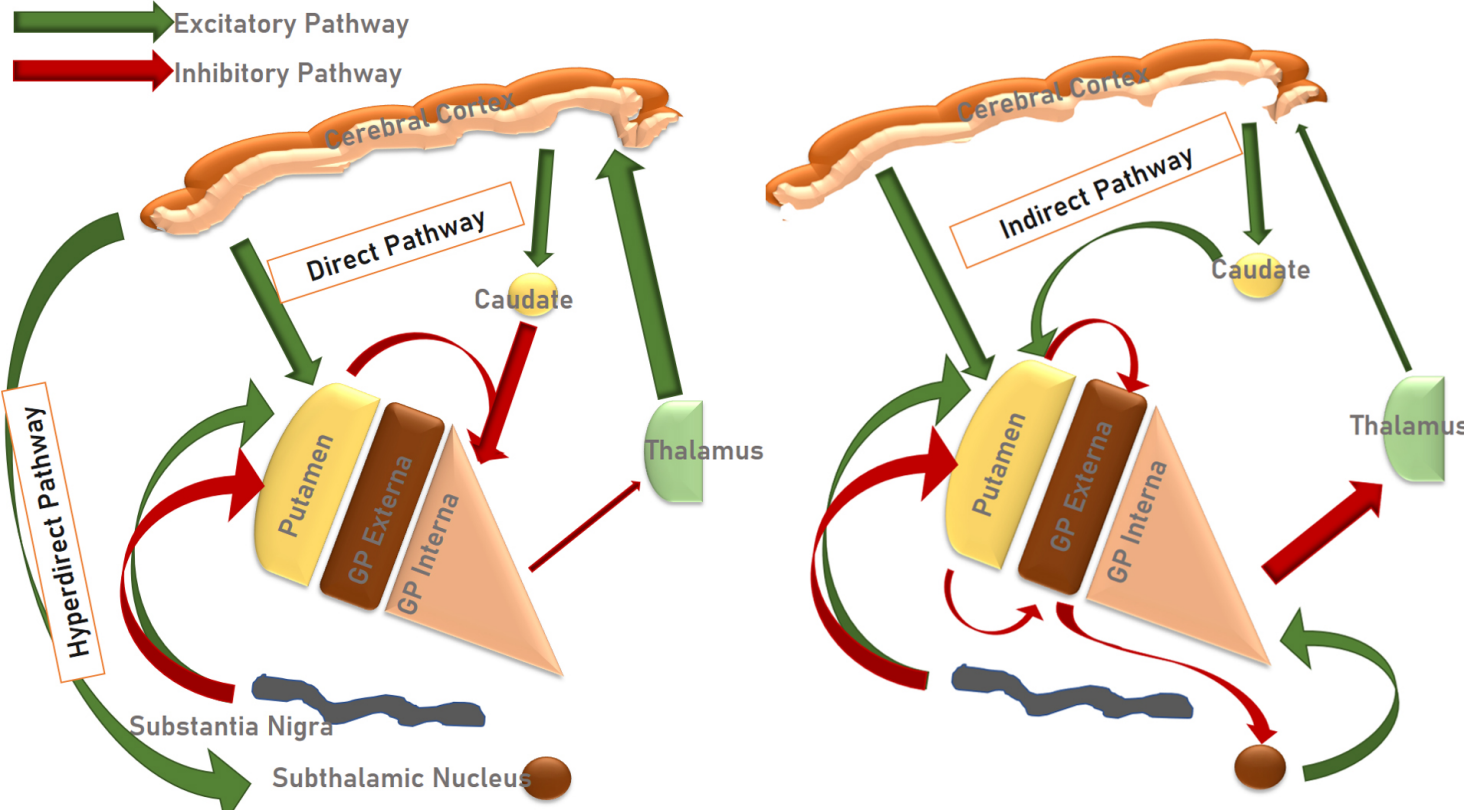
A schematic diagram detailing the normal inhibitory and excitatory pathways of the normal functioning indirect, hyperdirect, and direct pathways of the basal ganglia.

**Fig. (2):**
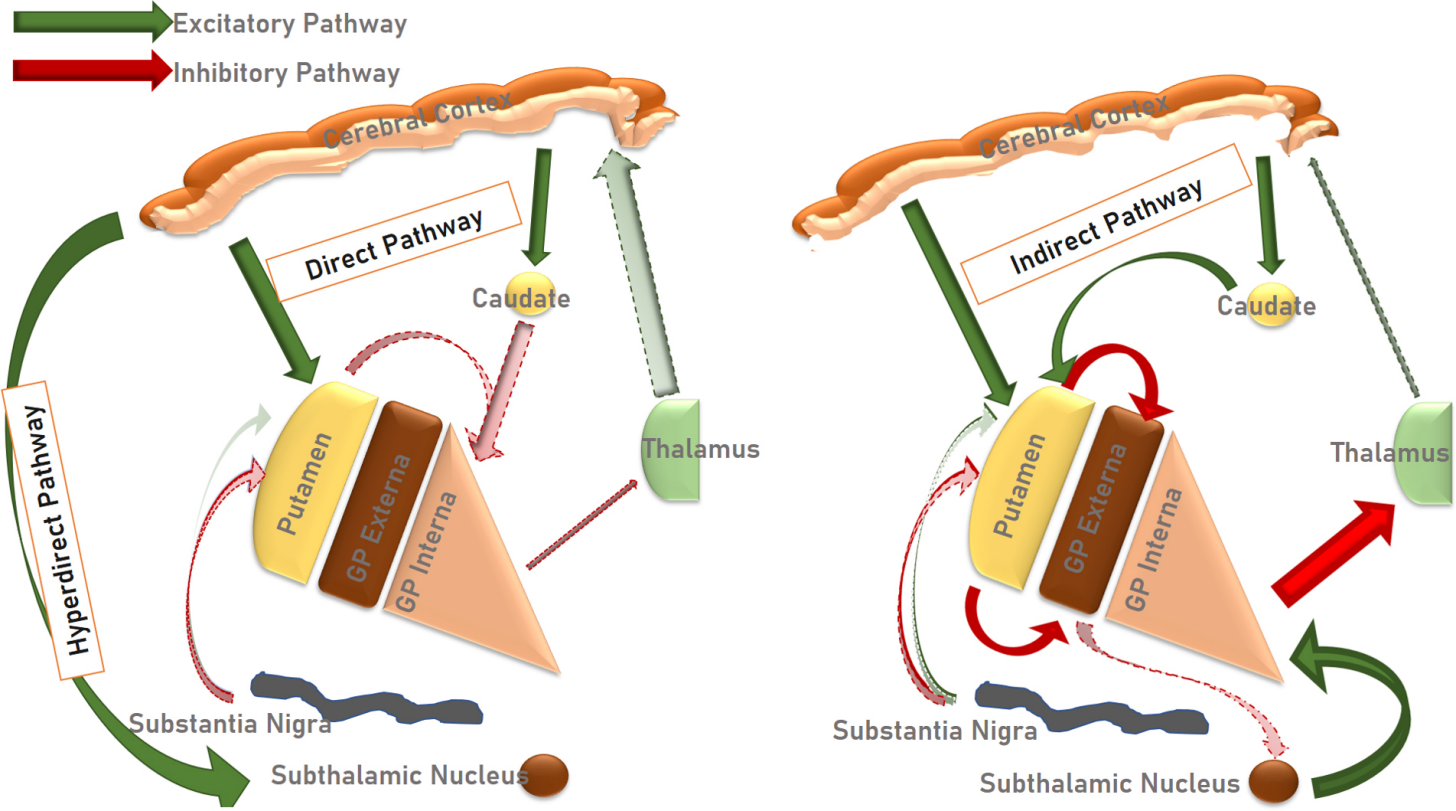
A schematic diagram detailing the pathological circuitry arising as a result of, among others, the dopaminergic degeneration of the substantia nigra, associated with Parkinson’s Disease.

As previously described, the striatum serves as the primary input nuclei of the basal ganglia, including dopaminergic projections from the SN. In turn, the striatum sends inhibitory output to both the GPe and the GPi. The neurons projecting to the GPi contain GABA and substance-P, and are positive for dopamine D1 receptors. The GPe receives GABAergic/enkephalinergic neurons, positive for dopamine D2 receptors. The cortico-striatal-GPi pathway is known as the ‘direct’, while the cortico-striatal-GPe-STN-GPi pathway is known as the ‘indirect’ pathway [7]. Briefly, activation of the direct pathway means that a glutamatergic signal acts on the striatum, increasing GABAergic inhibition of the GPi. The Gpi, in turn, ceases tonic inhibition of the ventral anterior and ventral lateral (VA-VL) parts of the thalamus, with the net result being increased motor cortical activity. On the other hand, activation of the indirect pathway entails glutamatergic activation of the striatum, increasing inhibitory signals to the GPe. This, in turn, disinhibits GABAergic inhibition of the GPi, resulting in increased tonic inhibition from the GPi to the thalamus and a decrease in motor signals. In all this, the dopaminergic projections from the SN play a modulatory role. In addition to the circuitry described above, there is the so-called ‘hyperdirect’ pathway, in which the motor cortex sends activating projections directly to the STN, in turn activating both the GPi, GPe and SN [7-12]. This scheme, while simple to grasp, has, however, been challenged by recent evidence, implying that the functional connectivity is more complicated than indicated here (Figs. **1** and **2**) [13].

## 4 PATHOPHYSIOLOGY OF PD IN RELATION TO SURGICAL TREATMENT

The most well-known pathological hallmark of PD is the loss of dopaminergic projection neurons in the SNr. This deficiency of dopamine transmission causes loss of striatal excitation, which in turn causes disinhibition of the GPi and STN. As these structures deliver inhibitory signals to the thalamus, the result is an overall decrease in the activity of thalamocortical transmission. Clinically, this translates to akinesia and rigidity [14]. Furthermore, with the loss of SN-dopaminergic activity affecting the STN, the result, according to a number of neurophysiological investigations, is the occurrence of an increased beta-band activity (approximately 13-30 Hz) and abnormally synchronized basal ganglia oscillatory activity causing both parkinsonism and dyskinesia, which are suppressed by STN DBS [15, 16]. Importantly, however, there is robust evidence implicating other neurotransmitter systems, such as the noradrenergic system for non-motor symptoms of PD [17] and the cholinergic system for gait problems and freezing of gait [18], perhaps in part explaining the poor effects of DBS on these different symptoms.

## 5 LESION THERAPIES

The very first surgeries targeting PD were different forms of ablation or surgical lesion interventions of either subcortical structures of the brain or spinal cord. The objective of these surgeries was mostly the control of tremor. Surgeries of the spinal cord included the unilateral pyradotomy, also known as Putnam procedure, performed as late as the 1950s [19]. This treatment involved a transection of the lateral portion of the pyramidal tract ipsilateral to the more severe tremor. Expanding upon the lateral pyradotomy, Ebin’s procedure consisted of transecting the pyramidal tract ipsilateral to tremor as well as the ventral part on the contralateral side. A final procedure was the complete transection of spinal cord white matter, ipsilateral to tremor. While some patients were able to achieve stable tremor control from these heroic measures, in many patients, the tremor would reappear after some time. Furthermore, the side effects were overall quite severe and included paresis of the affected side as well as a high rate of mortality.

In addition to the spinal transection surgeries for tremor, others attempted to alleviate symptoms of PD by resecting paravertebral sympathetic ganglia [20]; however, the results of this procedure were transient and the rate of adverse events was high.

Early cerebral lesioning therapies include Meyers’ attempts at controlling tremor and rigidity by explantation of the head of the caudate nucleus as well as the severing of the anterior portion of the internal capsule [21]. The transient nature of the alleviating effects of these procedures, however, led Meyers to conclude transection of the pallidofugal fibers to be the optimal treatment modality in PD, despite a perioperative mortality rate of almost 16% [22]. Other early targets of surgical alleviation were the pallidum proper, the thalamus, and the subthalamus or Forels H field.

For the most part, lesioning therapies have fallen out of favor as they have become superseded, first by the introduction of levodopa therapy, and recently by more advanced, targeting surgical therapies, such as DBS or MR guided focused ultrasound (MRgFUS) thalamotomy.

## 6 DEEP BRAIN STIMULATION

Since DBS was introduced, surgery has returned as a viable treatment modality for PD as well as other conditions manifesting with tremor. DBS was introduced as a treatment for PD in the 1990s and has since been recognized as a suitable treatment, especially for motor fluctuations, levodopa-induced dyskinesias, and tremor. The two most commonly used targets for DBS in PD are the GPi and the STN.

While there is still considerable debate regarding the precise mechanism of action of DBS, its clinical effects on the motor symptoms of PD, even in less advanced cases [23], are very well-established and, therefore, will not be discussed in this review. The improvement in activities of daily living and quality of life, along with the improvement of motor symptoms, is also uncontroversial [24]. A recent longitudinal study has shown that the subjective effects of DBS are generally favorable in the long term, allowing many individuals to maintain activities of daily living through a follow-up greater than 10 years. 73% of the patients included also reported an improved control of tremor for about 13 years [25]. On the other hand, there are also drawbacks to consider, such as neuropsychiatric side effects [26] and speech difficulties [27]. Taken together, these potential benefits and drawbacks mean careful consideration, and weighting of factors is required before a patient is referred for surgery.

### 6.1 Who is DBS Meant for?

There are several important factors to consider when choosing DBS surgery for the treatment of PD. Although no screening tool has yet reached the status of gold standard, several attempts have been made. Among these are the Core Assessment Program for Surgical Interventional Therapies in PD (CAPSIT-PD) [28], the RAND/UCLA [29], the FLASHQ-PD [30] and the Stimulus protocol [31]. However, there are still considerable caveats to applying these protocols [32]. Furthermore, when deciding to refer patients for DBS, there are several factors to take into consideration. Referring newly diagnosed patients for DBS runs the risk of overlooking atypical Parkinsonian syndromes. While there is no hard line as to when a patient is suitable, a general rule of 5 years of symptom duration is frequently used. Additionally, patients should have a recent assessment of levodopa responsiveness. As DBS is primarily a treatment for motor fluctuations, it is paramount that patients receive the optimal pharmacological therapy. Finally, a complete assessment of gait, posture, balance, and non-motor symptoms, and a careful genotypic and phenotypic characterization should also be performed (Table **1**) [33].

### 6.2 Comparing STN DBS and GPi DBS

Currently, the main targets of DBS surgery for PD are either the STN or the GPi.

There are no universally adopted guidelines for the selection of DBS targets in PD. There are, indeed, various factors to consider when deciding between the STN and the GPi. The general opinion appears to be that the STN is a superior target for the control of bradykinesia. However, the GPi might be slightly more advantageous for controlling dyskinesia [34]. Two systematic reviews on randomized controlled trials summarizing the differentiated effects of GPi and STN stimulation have been published [35, 36] as well as three additional systematic reviews comparing GPi and STN [37-39]. The main findings of the trials and reviews are summarized in Table **1**. The main findings were that GPi and STN were both suitable targets for tremor suppression; however, after 24 months, the analysis tended to favor STN.

In recent years, a significant amount of evidence comparing STN and GPi DBS directly has accumulated. The results of these studies as well as the findings of several reviews will be reviewed here. Weaver and colleagues [40] conducted a large-scale trial of DBS *versus* best medical therapy (BMT) in which patients in the DBS group were randomized 1:1 to either STN or GPi DBS. In a subsequent analysis of data collected at follow-up 36 months post-surgery, the efficacy of the two targets was compared [41]. In both groups (89 GPi DBS patients and 70 STN DBS patients), motor function, as measured by the UPDRS-III scores, improved between baseline and 36 months of both ON and OFF medication. Improvements were similar between targets and stable over time. Conversely, measures of neurocognitive function showed a gradual decline over time, and the Mattis Dementia Rating Scale scores declined faster for STN than GPi patients (*p* = 0.01). Depression scores did not change from baseline over 36 months, and no target differences were noted. Finally, quality of life and activities of daily living improved at 6 months on all subscales, but the improvement diminished over time for both DBS targets, possibly reflecting the progression of the underlying PD.

A similarly designed trial, the subthalamic nucleus *versus* globus pallidus bilateral deep brain stimulation for advanced Parkinson's disease (NSTAPS), investigated the effect of DBS at 12 [42] and at 36 [43] months in a group of 128 patients who, despite optimal medical treatment, experienced severe fluctuations, dyskinesias or painful dystonias. Patients were randomized 1:1 to receive either GPi or STN DBS. At 12 months, the primary outcome was the difference from baseline in disability assessed by the Academic Medical Center Linear Disability Scale (ALDS) and the composite score of cognitive, mood and behavioral effects. Secondary outcomes were changes in the UPDRS as well as the clinical dystonia rating scale (CDRS). In terms of the primary outcomes of the study, the authors found no significant difference between the two targets. However, for the UPDRS-III, STN DBS appeared to improve off-medication symptoms to a higher degree than GPi DBS. There were no significant differences between targets in measures of mood, cognition or behavior. At 36 months, the primary outcome measures were the UPDRS-III and the composite score of cognitive, mood and behavioral side effects assessment. STN was still favored in terms of the motor component of the UPDRS (33 *vs*. 28, *p* = 0.04). However, for the composite score, no significant change was detected between the two targets. In general, side effects incurred over the course of DBS treatment, apart from those sustained during an injury, which may be generally attributed to the proximity of target structures to other subcortical structures. While this may not be the entire explanation, what is clear is that for STN DBS, commonly, patients experience a decrease in verbal fluency as well as impaired memory [44].

The Deep-Brain Stimulation for Parkinson’s Disease Study Group compared GPi with STN DBS at six months [45] and at a final follow-up five to six years post-surgery [46]. At three months, patients were evaluated in a double-blinded, crossover design in which the immediate effects of stimulation were assessed using the UPDRS-III as the primary outcome. Participants were not randomized according to target, thus making direct comparisons between the effects difficult in this trial. However, corroborating the finding from the NSTAPS trial, the effect of STN DBS on motor symptoms was larger than for GPi. This superiority was, however, only present in the OFF medication state (51.3% *vs*. 33.3% improvement, respectively) and not in the ON medication state (25.8% *vs*. 26.8% improvement, respectively). At five-six years, 51 patients were evaluated (n = 36 for STN and n = 15 for GPi DBS). Again, the magnitude of the effects on the UPDRS-III was larger for the STN group compared to GPi. However, fewer adverse events were reported in the GPi-DBS group (Table **2**).

### 6.3 Advancements in Preoperative Planning and DBS Lead Placement

Preoperative planning includes target localization using MRI. This should be performed with precision as this is not error-free, and for targets, such as the STN, measuring, on average, 3 x 6 x 4 mm, can have clinical implications. In general, a preoperative MRI is modified and fused with a database of CT scans, and the location of target nuclei can be estimated in relation to these prespecified maps of anatomy. Suitable landmarks include the anterior-posterior commissural line (AC-PC line), and the margins of the third ventricle or the internal capsule.

Intraoperative microelectrode recording (MER) as an adjunct to the procedure of target localization is used in some centers as an added precision measure. Based on the neuronal firing patterns encountered by the microelectrode, it is possible to correct the placement of the stimulator during surgery. However, the dimensions and location of the STN often deviate compared to MER measurements. Several studies have sought to quantify this discrepancy [47, 48] with one review finding the deviation from the planned target to range from 1 to 4 mm, based on physiologic recordings [49]. Furthermore, in one large registry-based study, the revision rate was found to be between 15 and 34%, in part due to leads being placed in the non-motor regions of the subcortical nuclei [50].

Clinical assessments of tremor, bradykinesia and rigidity during intraoperative stimulation are also performed in some DBS centers. However, like MER, these assessments require the patient to be awake during the preoperative stages and while the leads are being positioned, this can cause distress and discomfort in many patients. There is growing evidence to suggest that DBS surgery performed under general anesthesia is superior in terms of safety and tolerability for the patients [51, 52]. However, as others have pointed out, there are several caveats when considering the pros and cons of asleep DBS. For one, the experience level of the surgical team is of substantial importance [53].

Improving the precision of preoperative and/or intraoperative targeting could solve some of the issues mentioned above, shortening the time of surgery and reducing patients’ discomfort.

### 6.4 Advancements in Preoperative Imaging

With increasing technical proficiency and capability, several new options for targeting have emerged. A recent feasibility study has successfully attempted to map the GPi using a high-field 7T MRI scanner for pre-surgical MRI in 17 patients. The authors found that using 7T MRI, they were able to accurately parcellate and identify the motor subregion of the GPi as the optimal stimulation target in patients scheduled to undergo DBS surgery [54]. While these findings are very promising, it should be noted that 7T MRI scanners are expensive and are not widely available for clinical purposes.

Connectivity-based lead placement using diffusion tensor tractography (DTI) has also been used in order to optimize lead placement and the clinical effects of DBS. In one early DTI study, the subthalamo-cortico-ponto-cerebellar and dentatothalamic tracts were visualized, and their position with respect to the most clinically effective contact was evaluated in nine patients with implanted STN electrodes [55]. The authors found that the most effective contact in these patients was close to the dentatothalamic tract. They also found better postoperative tremor control in patients with electrodes closer to the dentatothalamic tract, although this did not reach statistical significance. These preliminary findings would suggest that the preoperative identification of the dentatothalamic tract with tractography might be relevant for target planning. In another study with nine PD patients, Avecillas-Chasin and colleagues used 3T MRI with a combination of DTI and FLAIR sequences to parcellate the motor subregions of the STN and the ventral intermediate nucleus (VIM) of the thalamus [56]. This parcellation has since been replicated by other teams, and the topographical organization of the GP in relation to DBS has also been described [57-60]. While the precise clinical importance of these findings is difficult to gauge at the present moment, there are indications that the clinical outcome of DBS in PD may improve with DTI-based targeting [61]. Randomized controlled trials are, however, still needed to verify this hypothesis.

### 6.5 Other Targets for DBS

Apart from the most common DBS targets, the STN and the GPi, other targets have been studied in relation to PD. DBS of the thalamic VIM, generally used for patients with essential tremor, is a well-established surgical approach for tremor-dominant PD, although it has no effect on bradykinesia and rigidity. Several trials have found a reduction in tremor sub-scores of the UPDRS-III to be between 67-83% immediately post-operatively. Importantly, the effect was sustained ten years post-surgery [62-64]. The VIM is located centrally in a motor pathway from the motor cortex through the thalamus and to the dentate gyrus of the contralateral cerebellum, the dentate-rubro-thalamic tract (DRT). Prevailing theory suggests that VIM DBS likely acts by disrupting pathological oscillations in this network [65]. To support this finding, a study of eight VIM DBS PD patients, measuring regional cerebral blood flow (rCBF) using ^15^O-PET, found a decrease in rCBF in the dentate nucleus contralateral to stimulation [66]. While the VIM is not the most common target for DBS, it is one of the primary targets for other therapies for tremor, in particular MRgFUS, as discussed below.

Apart from tremor, other symptom specific-targets have been proposed. In particular, the SNr and the pedunculopontine nucleus (PPN) have been proposed as DBS targets to improve axial symptoms and gait problems in patients with PD. Axial symptoms are common in PD, particularly in more advanced stages, and in general, are not improved, and sometimes even exacerbated, by STN DBS, although there is some evidence to suggest that this procedure might alleviate some postural symptoms, such as trunk flexion [67]. This effect, however, appears to be modest and dependent on patients’ characteristics [68]. One proposed explanation for the lack of the effect of STN DBS on axial symptoms is that this procedure facilitates motor control through the modulation of cortico-thalamo-spinal pathways. Conversely, axial symptoms are caused by dysfunction of mesencephalic locomotor pathways. Theoretically, DBS of the SNr could provide greater functional control of axial symptoms by stimulating nigropontine projections. Unfortunately, there is a dearth of experimental evidence to recommend SNr DBS as a viable addition to STN stimulation. In a cross-over double-blinded randomized trial, Weiss and colleagues [69] found a reduction in episodes of Freezing of Gait (FoG) in PD patients with combined STN and SNr stimulation compared to standard STN stimulation only. More recently, Horn and colleagues [70] found an improvement of variables of gait kinematics and a general reduction in FoG frequency following STN+SNr DBS; however, there was a tendency for the UPDRS-III score to be higher, that is to say, worse, in STN+SNr compared to STN alone. Additionally, in both studies, the results were highly variable, indicating an uneven treatment response.

The role of the PPN in locomotion and gait initiation as well as its involvement in PD has been the subject of several research investigations. Briefly, the PPN is the main nucleus of the anatomically poorly defined mesencephalic locomotor region (MLR), and a loss of neurons here, as PD progresses, is associated with progressively severe akinesia [71]. Several trials on the effect of PPN DBS, with or without concomitant STN DBS, on gait symptoms have been carried out. Individually, these trials have varying results, with some trials finding limited beneficial effect [72] on gait and others finding a substantial effect on FoG and other gait-related symptoms [73]. Recently, the Neuromodulation of Gait Working Group of the Movement Disorder Society has reported a clinical review of published PPN DBS studies [74]. They observed a large variation in clinical findings that could in part be related to the lack of a standardized implantation methodology and the great variability in the clinical methodology used between and within surgical centers. Despite this variability, they estimated the effect of PPN DBS to be a 30% reduction in instances of FoG, based on a subset of studies reporting scores from individual patients. However, there was minimal evidence that PPN DBS could clearly improve the quality of life in PD. Furthermore, no specific preoperative markers of PPN DBS efficacy were identified.

Other brain regions that have been considered potential DBS targets include the ventralis oralis anterior and ventralis oralis posterior (VOA and VOP) nuclei of the thalamus. One trial of combined VIM and VOP DBS in 18 patients found this to be a feasible method for tremor suppression [75]. Interestingly, this approach could also be beneficial in cases of dystonic tremor [76].

## 7 RECENT DEVELOPMENTS AND FUTURE DIRECTIONS OF DBS

### 7.1 Directional Leads

A recent advancement in DBS technology has been the development of radially segmented electrodes, which allows the current flow to be directed both in the vertical and the horizontal planes. Compared to conventional DBS leads, this new electrode design enables clinicians to modify the size and shape of the volume of tissue activated to achieve improved precision of stimulation, and thus cause fewer side effects.

Directional DBS leads are now routinely used in clinical settings but, so far, few studies have been performed using directional stimulation. The evidence, however, points towards a wider therapeutic window, and thus, better control of side effects with this approach [77-80]. One recent paper reported the results of a retrospective assessment of the use of directionality in PD patients with STN DBS programmed in routine clinical care at a single center. The authors found that 74% of 57 patients with directional DBS leads in the STN had been programmed on directional stimulation for their stable settings, suggesting that directional programming in these patients was preferred for routine clinical care. As expected, the medial and anterior segments were more commonly preferred for chronic stimulation, while the lateral segment was rarely selected, most likely to avoid capsular effects. Additionally, directional stimulation led to substantial improvements in therapeutic current strength, therapeutic window, and total electrical energy delivered compared to omnidirectional stimulation [81].

### 7.2 3D Visualization of DBS Leads

One of the more time-consuming aspects of DBS is programming the stimulation. Although several algorithms have been developed, this aspect of the procedure is usually performed using a trial-and-error approach. Various factors can influence this procedure; such as individual patient anatomy as well as the many ways in which the programming can be set. To ease this process, several novel approaches have been developed. One study [82] investigated the utility of software providing a 3D visualization of the location of the DBS electrode in the brain and stimulation field to improve DBS programming. The authors found that this approach was significantly shorter than the conventional trial-and-error approach. However, adjustments were required for the software-based programming to be comparable in efficacy to the traditional approach. Refinements to this approach could potentially lead to an effective reduction of the time of initial DBS programming and improved clinical outcome.

### 7.3 Closing the Loop

The standard for DBS treatment is a tonic stimulation applied to the target area. As described above, constant stimulation DBS (cDBS) is an effective treatment for a range of PD symptoms. However, this approach is unable to adapt to changes in patients’ symptoms, especially if these occur in a short time span. Furthermore, constant stimulation increases the risk of adverse events, such as speech impairment [83]. To overcome this issue, there is increasing interest in adaptive or Closed-Loop DBS (aDBS or CL-DBS). In contrast to cDBS, aDBS can be schematically represented by a tripartite modular system, consisting of a sensing module for recording a relevant biomarker or feedback variable, a control module that integrates the sensory information and updates the stimulation parameters, and a stimulation module under hierarchical control by the control module [84]. The two most well-studied signals to date are local field potentials (LFPs) and electrocorticography (ECoG). For PD, the recording of LFPs and ECoG represents a specific challenge, as the recording must take place while stimulation is on, requiring suitable strategies for artifact elimination.

Moving beyond an experimental setting, some industrial manufacturers of DBS leads have begun developing equipment suitable for incorporating lead measurements into the stimulation paradigm. One such system is the PERCEPT^TM^, developed by Medtronic. While no clinical studies have, to date, established the precise use of this feature, it is likely that capabilities, such as this, will be useful in developing personalized stimulation paradigms, based on the recording of patient-specific LFPs. It may, further, be useful in guiding the placement and programming of highly personalized directional fields in order to increase the clinical effects and minimize the rate of side effects.

## 8 MAGNETIC RESONANCE-GUIDED FOCUSED ULTRASOUND

### 8.1 Procedural Considerations

While the principle behind MRgFUS has been known since the 50s, this treatment modality did not become prominent until 2014, when it was tested as a treatment for essential tremor. Since then, the area of application for MRgFUS has expanded to include tremor-dominant PD as well as other neurological diseases [85]. The rationale behind MRgFUS thalamotomy (MRgFUSth) and the treatment effects are similar to the early thalamotomy and VIM-DBS. Although considered a form of stereotactical neurosurgery, it is minimally invasive. Just before the procedure, a stereotactic head frame is fixed to the patient’s head under local anaesthetia. The patient then lies down in an MRI scanner, and a semi-spherical helmet containing ultrasound-generating elements is placed over the patient's shaved scalp to perform a non-invasive thermal ablation of the thalamus. At the beginning of sonification, focused ultrasound waves are precisely delivered under image guidance to the VIM with increasing intensity in order to disrupt its function. The patient is continuously monitored throughout the procedure to assess changes in tremor. Once the full suppression of tremor in the contralateral limb is achieved without the occurrence of side effects, permanent thermal ablation is performed. The most common side effects of this procedure include numbness in the face or hand and gait disturbance. Most of these side effects are due to transient edematous swelling of the surrounding tissue. For this reason, most side effects subside as the swelling goes down.

### 8.2 Efficacy of MRgFUS in PD

Although still in its infancy and thus subject to evolving knowledge of best practice, preliminary evidence suggests that MRgFUS has potential in tremor-dominant PD. However, at the time of this writing, large-scale, rigorously designed studies are still lacking. To date, two randomized controlled trials have been conducted assessing the efficacy and safety of MRgFUS as a treatment modality in tremor-dominant PD.

The first trial [86] was double-blinded and sham-controlled. A total of 27 PD patients were randomized to either active VIM-targeted MRgFUSth (n=20) or sham treatment (n=7). Of these 27 individuals, 19 were available for follow-up at 1 year. The study included only tremor-dominant, medicine-refractory PD patients. Tremor dominance was defined based on the tremor sub-items of the UDPRS compared to other symptoms, and medicine refractory was defined as 900 mg LEDD being unable to control tremors or LEDD causing unacceptable side effects.

The primary clinical outcome of the trial was the difference between groups in the Chair Sit and Reach Test (CSRT) score in the ON medication state after three months of treatment. For safety, the primary outcome was the frequency of adverse and serious events related to the procedure in the MRgFUSth compared to the sham group at 1-year post-intervention. At three months, the CSRT score was reduced by 62% in the MRgFUSth group compared to 22% in the sham group. Setting an arbitrary outcome of 50% tremor reduction as a success criterion at 1 year, on an assumption of intention-to-treat, the success rate was 55% (11/20). There were no statistically significant differences between active and sham group in terms of adverse events related to the MRgFUSth treatment. In terms of the motor component of the UPDRS, the median improvement (interquartile range) was 8 points (0.5-11.0) in the active group *versus* 1.0 (-5.0-9.0) in the sham-treated group. This finding was not significant. However, it should be noted that both groups experienced adverse events related to MRI, such as numbness, headache and dizziness. An ancillary report was published based on the findings of this trial [87]. This study assessed the non-motor outcomes in 27 individuals included in this trial, namely changes in cognition as the primary outcome, and mood and quality of life as secondary outcomes. The time points were the same as in the main trial at 3 and 12 months. Cognition was assessed with a battery of memory and cognitive tests. Mood and Quality of Life were assessed by the Beck Depression Index (BDI-II), Beck Anxiety Inventory, Frontal Systems Behavior Scale (FrSBe), and PDQ-39. Interestingly, the authors reportede no change in cognition, mood and quality of life at three months in the active group compared to the sham treatment group.

In another trial by Martinez-Fernandez [88], 40 patients were enrolled and assigned to either MRgFUS of the STN or a sham procedure in a 2:1 paradigm (27/13). Participants in this study were selected according to symptom asymmetry. This was defined as the ratio of the motor scores according to the MDS-UPDRS-III, being larger than 1.5 when comparing the most affected side with the least affected one. Further, symptoms had to be poorly controlled by dopaminergic medications, as assessed by four of the investigators. The main exclusion criteria were axial symptoms and a Hoehn and Yahr score of 2.5 or above. The primary efficacy outcome was the improvement in MDS-UPDRS-III score on the most affected side in the MRgFUS group compared to the control group at four months. The primary safety outcome was the incidence and severity of adverse events relating to the procedure at four months. At four months, the difference in motor score improvement on the most affected side between the two groups was 8.1 (6.0-10.3, *p* < 0.0001), suggesting a beneficial effect of this procedure.

### 8.3 Other Targets for MRgFUS

The VIM and STN are not the only nuclei investigated as potential targets for MRgFUS. Several trials evaluating the effect of focus ultrasound applied to the GPi have been published. However, none of these were randomized clinical trials. One trial, including 8 patients treated with MRgFUS of the GPi, found a decrease in UPDRS-III of 20.6 points OFF medication and 7.4 points ON medication after six months of treatment (no confidence interval reported) [89]. Another study was performed to investigate the effects of unilateral GPi MRgFUS on levodopa-induced dyskinesia and included 20 PD patients from two centers [90]. Compared to baseline, after three months of treatment, there was a 59% decrease in dyskinesia as measured by the self-reported items of the Unified Dyskinesia Rating Scale (*p* < 0.0001). The authors also reported a 44% OFF medication improvement in the MDS-UPDRS-III sub-score related to limbs of the treated side (*p* < 0.0001). The improvement in dyskinesia and motor score was sustained after 12 months of treatment. While promising, these results need to be replicated in larger randomized clinical trials.

## 9 OTHER FORMS OF NEUROMODULATORY SURGERY

Spinal cord stimulation (SCS) of the medial lemniscal pathway, an established therapy to treat chronic back and neuropathic pain, is currently being investigated as a treatment for gait problems in PD. Following the initial observation that SCS implanted for back pain in patients with PD also led to an improvement of their gait function [91], several promising case reports and series have shown a decrease in gait symptoms and overall decreased risk of falls, also in PD patients without back pain [92-95]. However, the findings of these unblinded and uncontrolled trials are variable, with a reported reduction in gait symptom severity ranging from 26% to 71%; one study showed a limited to no clinical effect [96]. Therefore, larger studies are required before SCS can be used more widely in PD. At the time of this writing, a single-center double-blind, placebo-controlled randomized trial with combined clinical and neuroimaging assessments [97] is being carried out to better understand the effects of SCS in PD patients with gait problems and investigate possible mechanisms of action of this procedure.

## 10 GENERAL CONSIDERATIONS FOR SURGERY IN PD

Over the last two decades, DBS surgery has shown its important role in the treatment of PD, offering a suitable approach to control levodopa-induced motor complications and dopamine-resistant symptoms. This has been accompanied by significant technological advancements that are already available in clinical settings or in advanced stages of development.

## CONCLUSION

Better targeting strategies, closed-loop DBS, and DBS lead-based recordings promise to deliver a more personalized DBS therapy, thus increasing efficacy and minimizing the side effects. Other surgical approaches, including MRgFUS and SCS, are also emerging, showing excellent preliminary results.

## Figures and Tables

**Table 1:** A comparison of some of the proposed guidelines for referral for DBS treatment.

**Protocol**	**Assessment Domain**					
**Flashq-PD**	**Base Data** ✓ PD likely ✓ Secondary PD unlikely	-	-	**OFF** ✓ L-Dopa ✓ Dyskinesia and dystonia rating **ON** ✓ Freezing ✓ Postural instability	**Cognitive** ✓ Memory Impairment ✓ Depression ✓ Psychosis	**Other** ✓ Swallowing function ✓ Urinary continency ✓ Treatment with blood thinning agent
**Rand/** **UCLA**	**Base Data** ✓ Age ✓ Disease duration	-	-	**OFF** ✓ UPDRS ✓ Hoehn & Yahr ✓ L-Dopa test	**Cognitive** ✓ Intellectual impairment	-
**Capsit-PD**	**Base Data** ✓ Age ✓ Disease duration	**Diary Containing** ✓ 30 min/day for 1 week ✓ Falls ✓ Freezing	**QoL** ✓ SF-36 ✓ QoL-PD	**OFF and ON** ✓ UPDRS ✓ Hoehn & Yahr ✓ L-Dopa test ✓ Hand/arm ✓ Walk ✓ Dyskinesia and Dystonia rating	**Cognitive** ✓ MATTIS ✓ MADRS ✓ Verbal fluency ✓ Face Auditory Serial Addition test ✓ Odd Man out Test ✓ Modified Brown Petersen Paradigm ✓ Rey Auditory Verbal Learning Test ✓ Visual Mnesic Battery of Signoret ✓ Tower of Hanoi	-

**Table 2:** Description of studies comparing STN and GPi as targets for Deep Brain Stimulation in Parkinson’s disease.

**First Author**	**Year**	**Study Design**	**Number of ** **Participants/** **Inclusions**	**Findings**	**Narrative Summary**
Anderson *et al.*	2005	Randomized, parallel blinded study	23 (20 at follow-up)	Both GPi and STN DBS improve medication OFF motor scores	• Randomized to GPi or STN DBS • They were assessed at baseline and follow-up • OFF medication motor improvement 39% in GPi • OFF medication motor improvement 48% in STN
Wong *et al.*	2020	Retrospective review of outcome	88 (57 STN and 31 GPi)	Both GPi and STN DBS decrease action- and rest tremor	• Retrospective assessment of STN or GPi DBS • At 6 months, STN was superior in reducing action tremor • Effect was not present at 12 months.
Weaver *et al.*	2009	Randomized, assessor blinded, controlled trial	235 (60 STN DBS, 61 GPi DBS and 134 BMT)	DBS was superior to BMT in achieving maximal ON time	• Randomized trial • Six months follow up • DBS patients, regardless of targeting better symptom control at six months • More ON time at 6 months • Improvement in quality of life in the DBS group.
Odekerken *et al.*	2013 & 2016	Randomized controlled trial	128 (65 GPi DBS and 63 STN DBS)	STN was slightly superior to GPi	• Randomized trial of GPi or STN DBS • Follow up after one and three years • No significant difference at one year • OFF medication symptom control, superior in the STN DBS group • Findings were persistent at three years
Obeso *et al.*	2001	Clinical trial	134 (96 STN DBS and 38 GPi DBS)	STN slightly preferred at six months compared to GPi	• Comparative study of DBS of the STN or the GPi • At six months, STN DBS had better symptom control • At six months, STN had a larger medication reduction
Moro *et al.*	2010	Open, non-randomized and later double-blind and randomized, prospective multicenter clinical trial	51 (35 with STN DBS and 16 with GPi DBS)	STN DBS showed larger symptom reduction, GPi DBS had fewer adverse events	• Mixed design with both • Follow-up after 5-6 years • STN DBS, larger effects on motor symptom severity • STN DBS larger improvement in activities of daily living and subjective quality of life. • GPi DBS associated with fewer adverse events.
Liu *et al.*	2019	Systematic review and meta-analysis	8 studies (822 patients)	GPi superior to STN to reduce dyskinesia at 12 months	• Review of differentiated effects on dyskinesias. • GPi DB better at controlling dyskinesias • STN DBS had a larger reduction in medication
Mao *et al.*	2019	Systematic review and Network meta-analysis	16 studies (1252 patients)	Both GPi and STN similarly improve UPDRS motor symptoms	• Comparison of several targets • GPi and STN similarly suited for symptom reduction • VIM better for tremor dominant PD
Xu *et al.*	2017	Systematic review and meta-analysis	8 studies (522 patients)	STN favored for OFF medication symptom control	• Review of differences in ON and OFF • STN DBS found more favorable • STN DBS led to larger reductions in medication
Mansouri *et al.*	2018	Systematic review and meta-analysis	13 studies	GPi and STN DBS similarly improve UPDRS motor scores at 36 months	• Review of the relative efficacy of STN and GPi DBS • Longest follow-up was 36 months • GPi and STN DBS comparable in improving motor scores. • Medication use less in STN DBS cohort • Depression scores lower in GPi cohort
Xie *et al.*	2016	Systematic review and meta-analysis	16 studies, 2186 participants	GPi and STN DBS similarly improve UPDRS motor scores	• Review of randomized trials of STN, GPi DBS and BMT

**Abbreviations:** STN = Subthalamic nucleus, GPi = Globus pallidus internus, DBS = Deep brain stimulation, UPDRS = Unified Parkinsons Disease Rating Scale, PDQ39 = Parkinsons Disease Questionnaire.

## LIST OF ABBREVIATIONS

ALDS: Academic Medical Center Linear Disability Scale
BMT: Best Medical Therapy
CRDS: Clinical Dystonia Rating Scale
(C)ZI: (Caudal) Zona Incerta
DBS: Deep Brain Stimulation
DRT: Dentato-Rubro-Thalamic tract
DTI: Diffusion Tensor Imaging
FLAIR: Fluid-Attenuated Inversion Recovery
FOG: Freezing of Gait
GABA: Gamma-amino-butyric acid
GPe: Globus Pallidus Externa
GPi: Globus Pallidus Interna
MDS: Movement Disorder Society
MER: Micro Electrode Recording
MLR: Mesencephalic Locomotor Region
MRgFUS: Magnetic Resonance Imaging Guided Focused Ultrasound
PD: Parkinson’s Disease
PPN: Pedunculopontine Nucleus
rCBF: Regional Cerebral Blood Flow
SN: Substantia Nigra
UPDRS: Unified Parkinson’s Disease Rating Scale
VA-VL: Ventral Anterior – Ventral Lateral
VIM: Ventral-Intermediate Nucleus
